# Duplex high resolution melting analysis (dHRMA) to detect two hot spot CYP24A1 pathogenic variants (PVs) associated to idiopathic infantile hypercalcemia (IIH)

**DOI:** 10.1007/s11033-021-06324-x

**Published:** 2021-04-17

**Authors:** Maria De Bonis, Elisa De Paolis, Maria Elisabetta Onori, Giorgia Mazzuccato, Antonio Gatto, Pietro Ferrara, Pietro Manuel Ferraro, Andrea Urbani, Angelo Minucci

**Affiliations:** 1 UOS Diagnostica Molecolare E Genomica, Fondazione Policlinico Universitario A. Gemelli-IRCCS, Largo Agostino Gemelli, 00168 Rome, Italy; 2 UOC Pediatria, Dipartimento Scienze della Salute della Donna, del Bambino E Di Sanità Pubblica- Area Salute del Bambino, Fondazione Policlinico Universitario A. Gemelli-IRCCS, Largo Agostino Gemelli, 00168 Rome, Italy; 3 UOC Nefrologia, Fondazione Policlinico Universitario A. Gemelli-IRCCS, Largo Agostino Gemelli, 00168 Rome, Italy; 4 Università Cattolica del Sacro Cuore, Largo Francesco Vito, 1, 00168 Rome, Italy

**Keywords:** Idiopathic Infantile Hypercalcemia, Duplex assay, High resolution melting analysis, *CYP24A1* gene

## Abstract

Pathogenic variants (PVs) in *CYP24A1* gene are associated with Idiopathic Infantile Hypercalcemia disease (IIH). The identification of *CYP24A1* PVs can be a useful tool for the improvement of target therapeutic strategies. Aim of this study is to set up a rapid and inexpensive High Resolution Melting Analysis (HRMA)-based method for the simultaneous genotyping of two *hot spot* PVs in *CYP24A1* gene, involved in IIH. A duplex-HRMA (dHRMA) was designed in order to detect simultaneously *CYP24A1 c.428_430delAAG*, p.(Glu143del) (rs777676129) and *c.1186C* > *T*, p.(Arg396Trp) (rs114368325), in peculiar cases addressed to our Laboratory. dHRMA was able to identify clearly and simultaneously both *hot spot CYP24A1* PVs evaluating melting curve shape and melting temperature (T_m_). This is the first dHRMA approach to rapidly screen the two most frequent *CYP24A1* PVs in peculiar case, providing useful information for diagnosis and patient management in IIH disease.

## Introduction

The term Idiopathic Infantile Hypercalcemia (IIH, OMIM 143880) first received attention almost 70 years ago in the UK, when symptomatic hypercalcemia was observed in infants after receiving high doses of vitamin D for the prevention of rickets [1, 2]

Pathogenic variants (PVs) in the *CYP24A1* gene, involved in the degradation of vitamin-D, have been identified as being a relevant part of the IIH etiology [3]. In particular, the clinical and biological role of *CYP24A1* gene*,* encoding the vitamin D-24-hydroxylase, lies in the metabolism of the 1,25(OH)_2_D, the physiologically active form of vitamin D. CYP24A1 enzyme is responsible of the 1,25(OH)_2_D catabolism and also it enhances the turnover and elimination of the 25(OH)D, the abundant precursor metabolite and storage form of vitamin D [4]. PVs in the *CYP24A1* gene can lead to elevated levels of 1,25(OH)_2_D, cause of pathological absorptive hypercalcemia and hypercalciuria. In the affected subjects, this condition may predispose to renal complications such as nephrocalcinosis and nephrolithiasis. Moreover, calcium deposition in mitochondrial structures, with a consequent damage of metabolism, may lead to renal epithelium impairment and tubular necrosis, potentially resulting in chronic kidney disease [5–7].

In this context, it is emphasized the importance of evaluation of *CYP24A1* gene as a crucial advanced diagnostic tool in the definitive diagnosis of IIH; furthermore, the *CYP24A1* PVs identification can be useful for the improvement of target therapeutic strategies, principally aimed to the control of calcium imbalance and the prevention of progression to chronic kidney disease.

## Case presentation

We report the case of a 6-years-old Italian male child with a personal history of bilateral nephrocalcinosis, severe hypercalcemia, increased urinary calcium/creatinine ratio and suppressed parathyroid hormone (PTH). The patient was born preterm at 32 weeks’ gestational age after an uncomplicated pregnancy and normal delivery with a birth weight of 2730 g.

At 10 months old he was hospitalized in order to observe his excessive failure to thrive, inadequate feeding and vomiting. Routine laboratory data showed: serum calcium levels of 16.7 mg/dl, 25-OH-Vitamin D 75.8 ng/ml and extremely low PTH serum levels (4.4 pg/ml). The first 24 h urine collection test revealed: calcium 72 mg/L, phosphorus 126 mg/L, creatinine 40 mg/L and urinary calcium/creatinine ratio of 1.8 mg/L. Abdominal ultrasound revealed bilateral nephrocalcinosis. Brain magnetic resonance imaging, echocardiogram, skeleton and chest X-Ray were normal. During hospitalization he started intravenous rehydration therapy with reduction of serum calcium (up to 12.6 mg/dl) so he was discharged with a low-calcium diet.

The patient referred to our pediatric nephrology ambulatory for the first time at 6 years old. Auxological data showed weight and stature in the normal range. His serum creatinine was 0.49 mg/dl (0.67–1.16 mg/dl), serum calcium 10.9 mg/dl (8.5–10.1 mg/dl), PTH is still low 2.7 pg/ml (14.0–72 pg/ml) and 25-OH-Vitamin D 46.7 ng/ml (31–100 ng/ml). Urinary calcium/creatinine ratio was 0.6 mg/L.

Under the suspicion of IIH, we performed molecular analysis of *CYP24A1* gene. The patient was firstly screened for the Italian *hot spot* variant *c.428_430delAAG* (rs777676129) by HRMA, accordingly with our previously published protocol [8]. At the melting profile evaluation, the sample resulted as negative for the targeted variant. Consecutively, we performed the sequencing of all coding and flanking intronic regions, as previously published [9]. Gene sequencing revealed an homozygous PV: the *c.1186C* > *T* (rs114368325), p.(Arg396Trp). This variant is reported by Gigante et al. [10] as *hot spot* PV in the Italian population, together with the *c.428_430delAAG* variant. These two PVs are detected in about 50% of all IIH Italian patients. Taking into account the literature data regarding the distribution of *CYP24A1* PVs, we decided to implement previous molecular diagnostic workflow of IIH [8], developing a duplex HRMA (dHRMA) for the simultaneous screening of these two Italian *hot spot CYP24A1* PVs.

## Materials and methods

### DNA extraction

After obtaining a written informed consent from patient’s parents, molecular analysis of *CYP24A1* gene was performed. Genomic DNA was extracted from peripheral blood leukocytes by an automatic DNA device (MagCore HF16 Plus, Diatech Lab Line, Jesi, Italy) with MagCore Genomic DNA Whole Blood Kit (RBC Bioscience Corp. TAIWAN). The DNA concentration and purity were tested using QFX Fluorometer (DeNovix Inc., Wilmington, USA) according to the manufacturer’s instructions.

### DNA sequencing

Samples were amplified using primers selected to cover all coding exons and flanking intronic regions of *CYP24A1*, as previously reported [9]. PCR products were bi-directionally sequenced using the BigDye® Terminator v3.1 Cycle Sequencing Kit on the Applied Biosystems 3500 Genetic Analyzer (Life Technologies, Carlsbad, CA, USA). The data were then analyzed with both SeqScape® software v.3 and Sequencing Analysis Software v.6 (Thermo Fisher Scientific).

### HRM design, optimization and conditions

To develop a duplex assay able to detect the two abovementioned variants, the design of new primers pair for the *c.1186C* > *T* variant was made taking into account the melting characteristics of the one already used in our laboratory for the *c.428_430delAAG* detection [8]. Particularly, in order to use the same annealing temperature (T_a_) we used the freely available software Primer3 (http://primer3.ut.ee/). The designed primers amplify an amplicon of 59 bp surrounding the *c.1186C* > *T* variant and have the following sequence: forward (F) 5′-AGGCTTACGCCGAGTGTAC-3′ and reverse (R) 5′-CCCAGAACTGTTGCCTTGTC-3′. In addition, to avoid overlapping between the two target amplicons, we used Oligo Calc software (biotools.nubic.northwestern.edu/OligoCalc.html) for melting temperature (T_m_) prediction. To optimize the test, we firstly performed the PCR-HRMA only for the *c.1186C* > *T* variant in order to verify: 1) the T_a_, 2) absence of aspecific products or primer’s dimer and finally 3) to confirm the predicted T_m_ of designed fragment. Subsequently, a duplex-PCR was performed to evaluate the amplification performance of both target amplicons. Consequently, we optimized the primers’ concentration to achieve a balanced amplification. After that, and once the parameters are set for each target, duplex PCR/HRMA was carried out using the LightCycler® 480 Real-Time PCR systems (Roche Molecular Systems, Inc.) on a 20 μL reaction mixture containing 2X LightCycler® 480 High-Resolution Melting Master (including buffer, Taq polymerase, nucleotides and proprietary ds-DNA saturating binding dye), 2.5 nM of MgCl_2_, 15 ng DNA template and primers. The rs777676129 and rs114368325 primers were used at final concentration of 0.15 and 0.30 μM, respectively.

PCR conditions included an initial hold at 95 °C for 10′ followed by 55 cycles with a denaturation step at 95 °C for 10′′ and a combined annealing/elongation step at 59 °C for 15″and 72 °C 10′, respectively. The melting program included denaturation at 95 °C for 1′, renaturation at 40 °C for 1′ and subsequent melting consisting of a continuous reading of fluorescent.

#### HRMA

Melting curve analysis was performed using the Gene Scanning Software (version 1.2, Roche Diagnostics) with “Gene scanning” and “T_m_ calling” tools. The normalization settings were the following: pre-melting normalization (76.42–77.37 °C), post-melting normalization (86.56–87.51 °C) and temperature shift with a threshold = 1. HRMA of both shape and peak height was performed in duplicate for each sample.

## Results

### HRMA

**Fig. 1:**
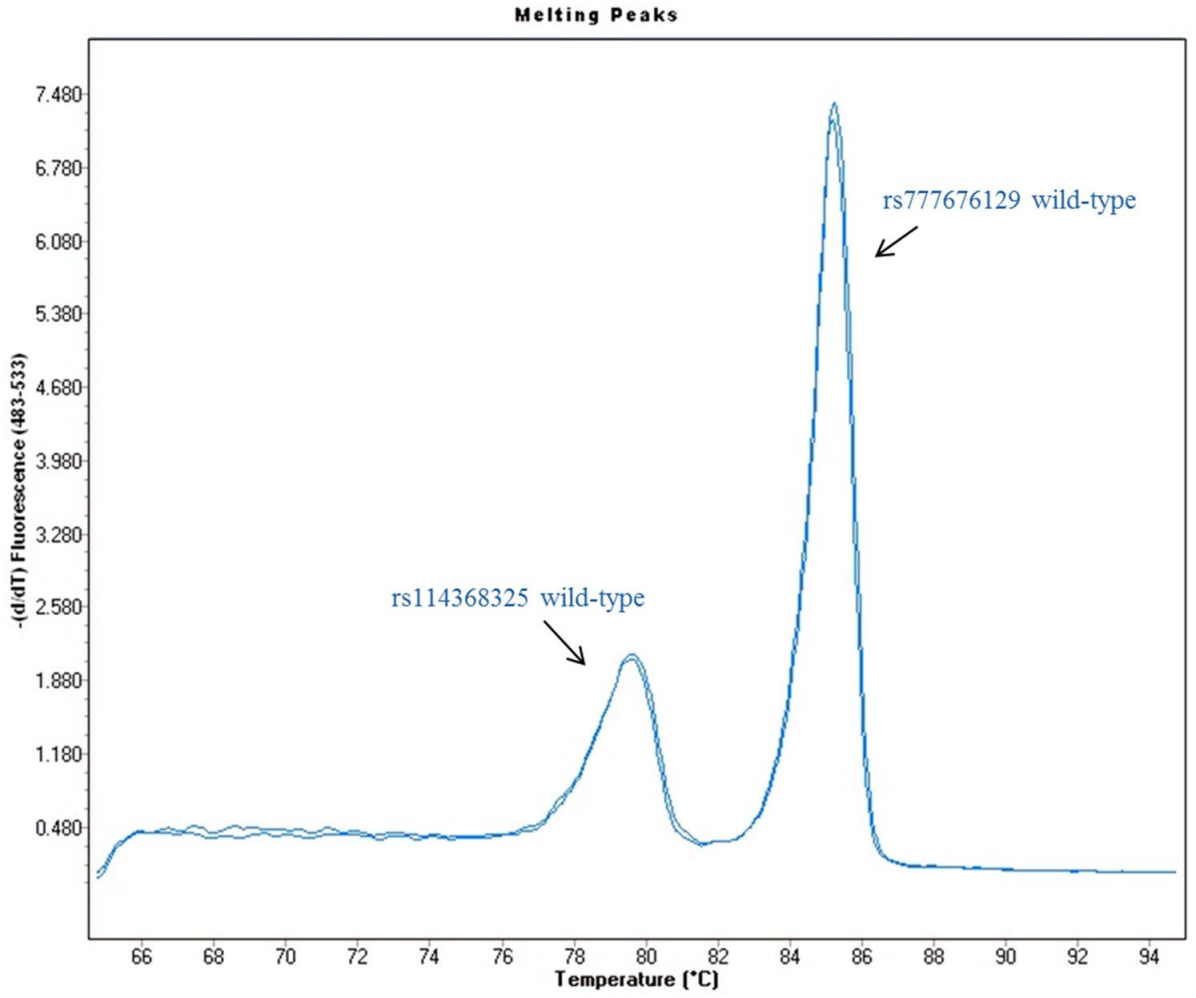
Melting peaks of the two wild-type amplicons analyzed for *CYP24A1*. The first peak represents the wild-type amplicon of rs114368325, instead the second one describes the wild-type amplicon of rs777676129

For HRMA set up, available samples with known *CYP24A1* status were used as positive and wild-type (wt) controls. In details, n = 2 samples with the *c.428_430delAAG* variant and n = 2 samples with the *c.1186C* > *T* variant (homozygous and heterozygous). Two samples with no *CYP24A1* variants were used as wt controls. Furthermore, we also included n = 1 compound heterozygous sample, carrying both variants tested. As above mentioned, the two sets of primers have been designed *ad hoc* to amplify two different target sequences and, consequently, each amplicon are indicative of only one common mutant site in *CYP24A1* gene. The T_m_ difference between the two wt amplicons was at least 6 °C and, consequently, the associated T_m_ profiles did not overlap (Fig. 1). Due to these melting characteristics and behavior, the two HRMA could be performed and analyzed at same time in a duplex assay.

**Fig. 2:**
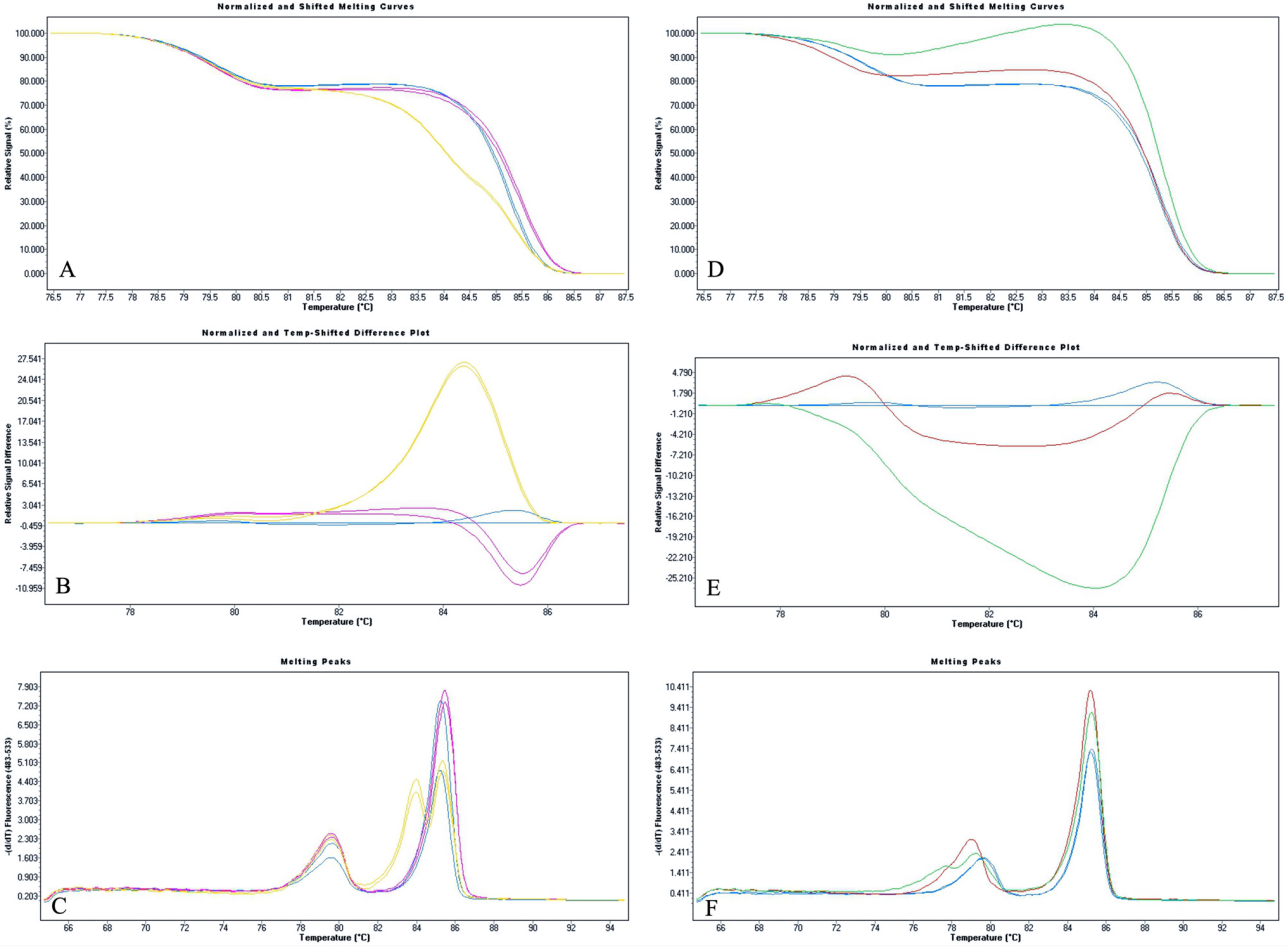
HRMA analysis of *CYP24A1* PVs rs777676129 and rs114368325. Homozygous and heterozygous profiles of *CYP24A1 c.428_430delAAG* (rs777676129) and *c.1186C* > *T* (rs114368325) compared with wild-type genotype are reported in panels from **a–c** and **d–f**, respectively. The three groups of panels show respectively: Normalized and Temperature-shifted Plot (**a, d**), Difference Plot (**b, e**) and Derivative Plot (**c, f**). Data were reported for wild-type (blue), heterozygous *c.428_430delAAG* (yellow), homozygous *c.428_430delAAG* (violet), heterozygous *c.1186C* > *T* (green) and homozygous *c.1186C* > *T* (red). (Color figure online)

**Fig. 3:**
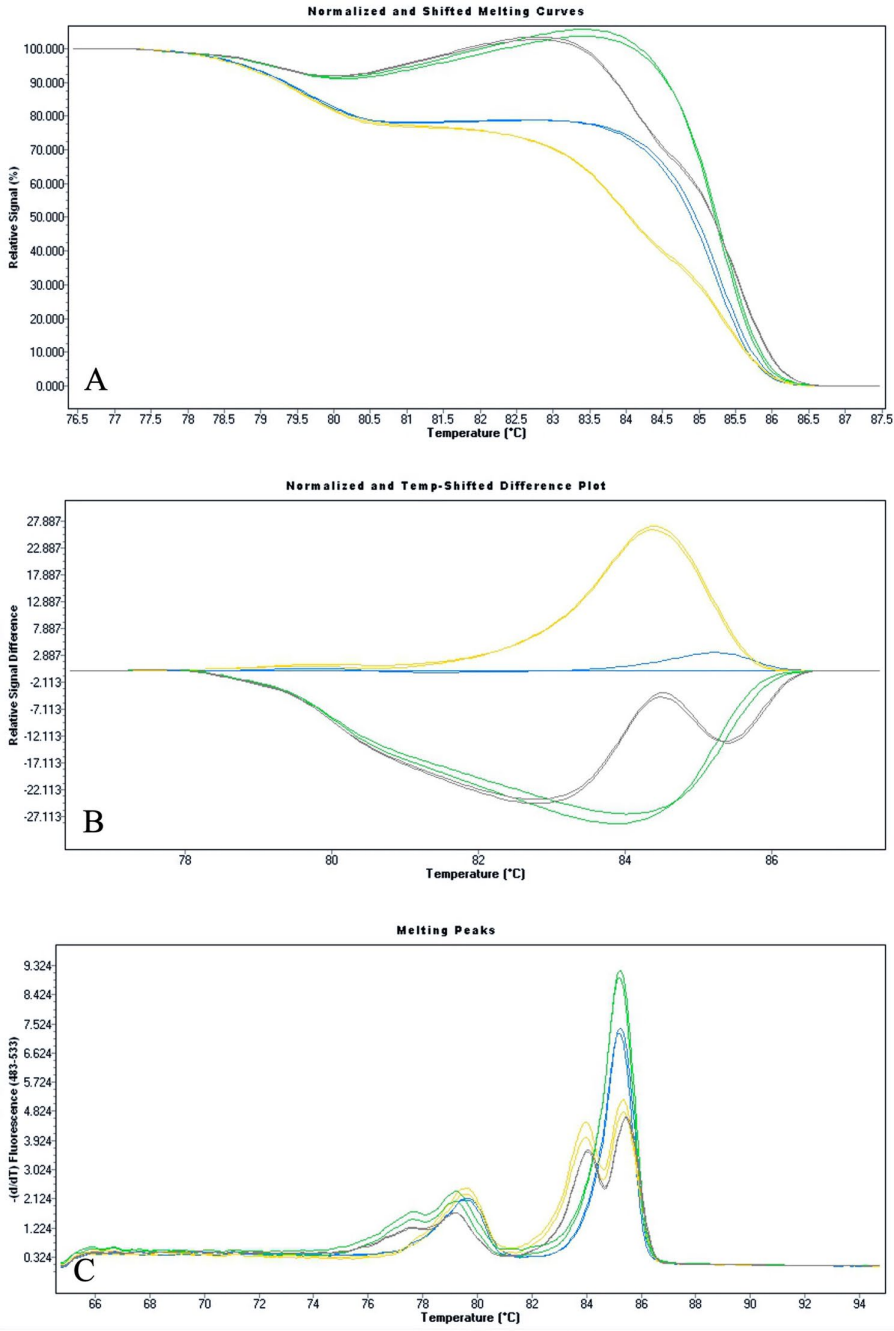
Normalized and Temperature-shifted Plot (**a**), Difference Plot (**b**) and Derivative Plot (**c**) of heterozygous *c.428_430delAAG* (yellow), heterozygous *c.1186C* > *T* (green), compound heterozygous *c.428_430delAAG/ c.1186C* > *T* (gray) compared with wild-type ones (blue). The compound heterozygous sample (gray profile) shows a melting curve behavior clearly distinguishable from the other heterozygous genotype of *CYP24A1* gene. (Color figure online)

**Fig. 4:**
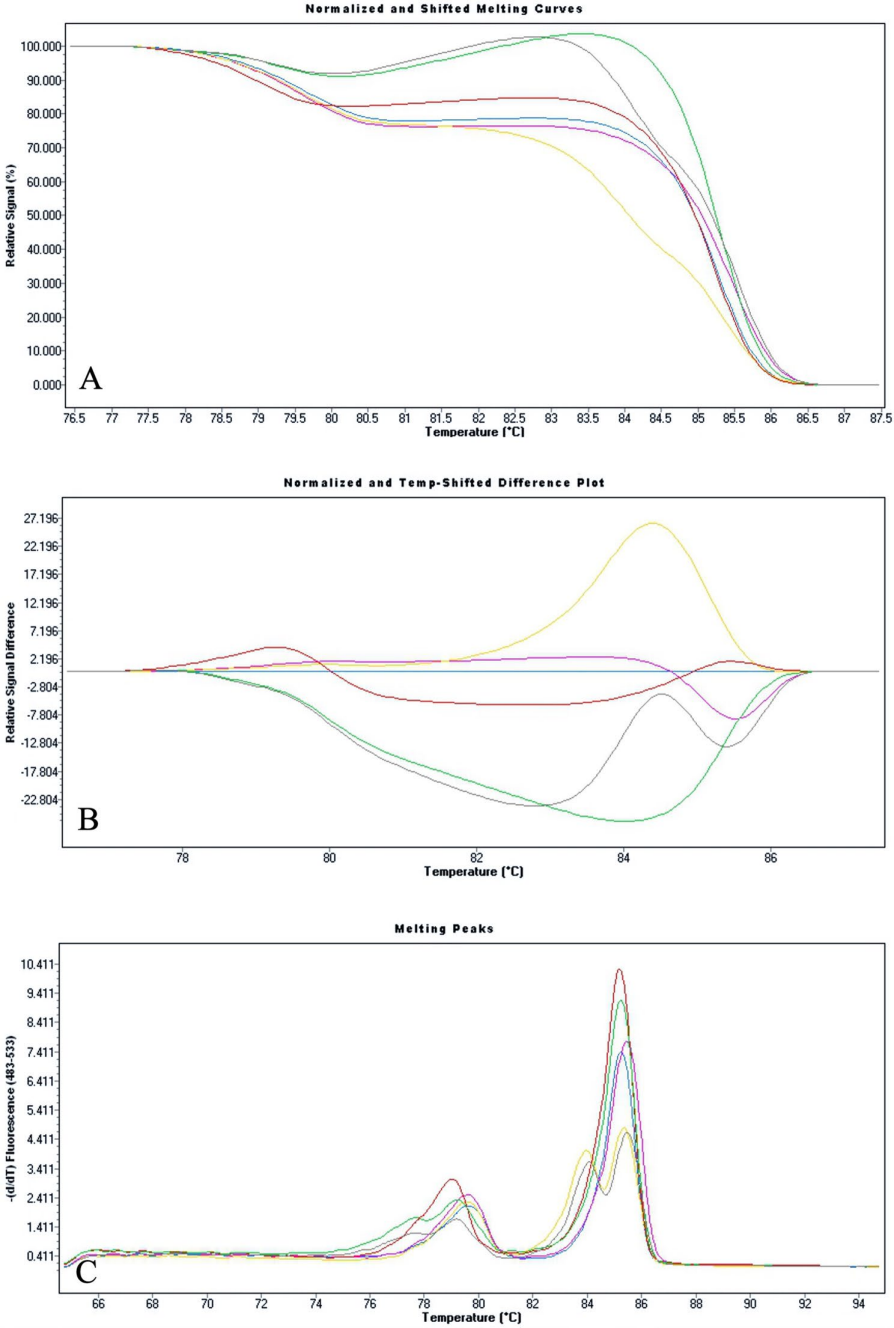
Normalized and Temperature-shifted Plot (**a**), Difference Plot (**b**) and Derivative Plot (**c**) of six genotypes tested in our duplex-PCR/HRMA assay. All genotypes were clearly distinguishable from each other

For all samples, the genotype assignment obtained by dHRMA was concordant with results of single plex HRMA and Sanger sequencing. In fact, we were able to clearly genotype all samples by evaluating their specific melting profiles along with the T_m_ values. The different *CYP24A1* genotypes exhibit a specific melting behavior, as observed in both *Normalized* and *Temperature-shifted* and *Different Plot* (Fig. 2). Because the T_ms_ of the two amplicons were clearly different, in each sample we can observe two different genotyping curves, each related to one locus. In detail, the melting peaks of rs777676129 amplicons were: 85.14 °C, 84.68 °C and 85.35 °C for wt, heterozygous and homozygous genotypes, respectively. The rs114368325 amplicons showed T_m_ of 79.37 °C, 78.69 °C and 78.81 °C of wt, heterozygous and homozygous genotypes, respectively. Furthermore, we were also able to identify the compound heterozygosis by evaluating its specific melting profile (Fig. 3) as well as the T_m_ shift compared to the wt ones (Table 1). As shown in Fig. 4, all genotypes were clearly distinguishable from wt sample.

**Table 1:** Genotypes and related T_m_

Genotype	T_m_ (°C) rs114368325 ± SD	T_m_ (°C) rs777676129 ± SD
Wild-type	79.37 ± 0.02	85.14 ± 0.02
Heterozygote *c.1186C* > *T*	78.69 ± 0.05	85.12 ± 0.01
Homozygote *c.1186C* > *T*	78.81 ± 0.03	85.07 ± 0.01
Heterozygote *c.428_430delAAG*	79.41 ± 0.01	84.68 ± 0.01
Homozygote *c.428_430delAAG*	79.43 ± 0.01	85.35 ± 0.02
Compound *c.1186C* > *T* / *c.428_430delAAG*	78.57 ± 0.02	84.90 ± 0.02

The average of temperature melting values of each genotype of both amplicons are reported. The T_ms_ of the all genotype’s amplicons are different form each other

Moreover, all genotypes obtained from the duplex HRMA were confirmed by sequencing.

### Validation and sensitivity of HRMA

To validate our protocol, duplex-PCR/HRMA was performed on further 10 DNA samples with the suspicion of IIH. Among of these, we found 1 heterozygous for the *c.428_430delAAG* and 1 heterozygous for the *c.1186C* > *T* variants*.* All HRMA results were verified by direct DNA sequencing, which confirmed all the genotypes, achieving 100% of concordance.

We evaluated the intra-assay precision analyzing 5 replicates of one single wild-type and homozygous/heterozygous samples, respectively. Furthermore, the inter-assay precision was assessed running the same wild-type, homozygous and heterozygous samples in triplicates on 3 different PCR/HRM experiments. We achieved a full superimposability and reproducibility of melting profiles and T_m_.

In order to test the sensitivity of HRMA, we evaluated serial dilutions (1:2, 1:4, 1:8, 1:16) of wt, homozygous and heterozygous DNAs, starting from 15 ng/μL. Melting curves of positive samples were still well differentiated as compared to wt ones, until the lowest variant allele frequency investigated (data not shown).

## Discussion

The patient described herein presents typical clinical signs of *CYP24A1* loss-of-function variants, as the *c.1186C* > *T,* an Italian *hot spot* PV [10]. The major strength of the current study was to set up an efficient and robust molecular test based on duplex assay coupled with HRMA to screen two Italian *hot spot* PVS in *CYP24A1* gene, implementing the previous our molecular diagnostic workflow for IIH disease [8]. This new approach was able to clearly discriminate the *c.428_430delAAG* and the *c.1186C* > *T* allele in either heterozygous or homozygous status, simultaneously.

Herein, HRMA strategy proves a flexible methodology that allowed us to develop a sensitive, cost-effective and less-laborious duplex HRM assay to unambiguous genotype the two most frequent *CYP24A1* PVs (rs777676129 and rs114368325) in Italian cohort simultaneously within a single tube. To the best of our knowledge, this is the first HRMA approach to rapidly screen the two most frequent *CYP24A1* PVs in peculiar cases with typical clinical signs of IIH disease.

IIH is a rare disease characterized by wide range of clinical signs as failure to thrive, dehydration, vomiting, nephrocalcinosis, and hypercalcemia. In the past, IIH was diagnosed after exclusion of other conditions related to development of hypercalcemia, such as Williams-Beuren syndrome, hyperparathyroidism, diuretic usage, and excess vitamin D intake [1, 2, 11]. Currently, PVs in *CYP24A1*, which encodes the enzyme 24-hydroxylase responsible for degradation of 25-hydroxyvitamin D (25-OH-D) and 1,25-dihydroxyvitamin D (1,25-OH-D), were associated with IIH. Since the identification that PVs in *CYP24A1* are responsible for IIH, many case reports have been reported, leading to an increased insight into clinical, biochemical and genetic characteristics of this disease [12]. To date, a total of 41 PVs associated with IIH were identified for the *CYP24A1*, emphasizing how *CYP24A1* genetic test was critical and essential for the final diagnosis of IIH. Nevertheless, many questions remain unanswered as the specific prevalence of the disease, the existence of a genotype–phenotype correlation and the best treatment of hypercalcemia [12, 13].

In the first Italian report, performed in a small cohort of patients, Gigante et al. [10]*.* describes 6 different *CYP24A1* mutation, including one small deletion (p.Glu143del), 4 missense mutations (p.Leu148Pro; p.Arg396Trp; p.Pro503 Leu; p.Glu383Gln) and one non sense mutation (p.Tyr220*) [10, 13]. Recently, Brancatella et al*.* [12], screened for *CYP24A1* mutations a large Italian family, reporting a nonsense *CYP24A1* gene mutation, the (p.Arg223*), previously described by two other research groups [6, 9]. Even if the exact frequencies of these mutations in a large cohort of IIH patients are not currently available, the *c.428_430delAAG* p.(Glu143del) (rs777676129) and the *c.1186C* > *T* p.(Arg396Trp) (rs114368325), detected in about 50% of all Italian patients, represents the two *hot spot* PVs in *CYP24A1* gene in an Italian cohort [10].

Given the high prevalence of these PVs in Italian cohort, the development of an efficient molecular screening test for *CYP24A1* represents an improvement of clinical laboratory routine.

Although Sanger sequencing and next-generation sequencing (NGS) are two of most common mutation detection methods for large-scale genomics sequencing samples, they can be expensive and time-consuming. Additionally, NGS method generates a large quantity of data, which is not always necessary; moreover, NGS results usually require validation and complex bioinformatics analysis for interpretation [14]. In this context, alternative method of analysis, as HRMA, might serve as a complementary approach for detecting the presence genetic variants. HRM is an efficient and rapid scanning method that can dramatically reduce the amount of sequencing. HRMA is highly suitable for the detection of single-base variants, deletions, or insertions [15–17]. In addition, HRMA offers several attractive advantages over other conventional gene scanning methods, such as no post-PCR processing steps, complete closed-tube format, and rapid turnaround time [18–21].

## Conclusions

In this study, HRMA has been successfully used to genotype the Italian *CYP24A1* PVs rs777676129 and rs114368325. This robust and simple molecular assay could be readily adopted by any genomic diagnostic laboratory with HRM capability, with the aim to screen rapidly the two most frequent *CYP24A1* PVs in peculiar case with typical signs of IIH disease.

The molecular diagnostic workflow, herein described, represents a first-line tool to identify simultaneously the two Italian PVs *hot spot* in patients with suspicion of IIH but doesn’t give a high risk of false negative results; in fact, if the samples result as negative for the targeted variants in the this first screening step, our workflow provides for sequencing of all coding and flanking intronic regions of *CYP24A1*.

Finally, this molecular approach could improve the diagnostic and clinical workflow of IIH patients, quickly identifying affected subjects, improving the patient management and supporting the clinicians for both diagnostic and therapeutic purpose.

## Abbreviations

IIH: Idiopathic infantile hypercalcemia
PVs: Pathogenic variants
PTH: Parathyroid hormone
HRMA: High resolution melting analysis
T_m_: Melting temperature
T_a_: Annealing temperature
NGS: Next generation sequencing
